# Supplementary material for: Assessment of Enterovirus Excretion and Identification of VDPVs in Patients with Primary Immunodeficiency in India: Outcome of ICMR–WHO Collaborative Study Phase-I
Source: Vaccines (Basel). 2023 Jul 6;11(7):1211. doi: 10.3390/vaccines11071211 (PMC10383878; doi:10.3390/vaccines11071211)
Supplement: Supplementary file 1 [file vaccines-11-01211-s001.zip › vaccines-2297611-supplementary.pdf]

Table S1. Enterovirus positive cases among different PID types in the Phase-I study.

PID Types	Total no. of cases	Total no. of cases positive for Enterovirus
CVID	20	6
Agammaglobulinemia	16	0
XLA	16	2 (1 P3SL)
Hypogammaglobulinemia	9	1
TRNT1 deficiency	2	2
IgA deficiency	1	0
PIK3CD	1	0
SCID	14	2 (1 P1SL)
WAS	12	5 (1 P3SL)
Hyper IgM syndrome	9	4 (1 iVDPV)
Dock 8 deficiency	6	1
AT with IgA deficiency	2	2
Ataxia Telangiectasia	2	2
CARD11 LOF	1	0
MHC Class II deficiency	1	0
ICF syndrome	2	1
CMC-STAT1 GOF	7	1
Leucocyte adhesion defect (LADI/III)	5	0
Chronic Granulomatous Disease (CGD)	3	0
HLH	3	0
MSMD	3	1
ALPS	2	0
Auto Inflammatory disease	2	1
SPENCDI	2	1
CARMIL-2	1	0
CD55 deficiency	1	0
Chediak Higashi Syndrome	1	1
Complement defects	1	0
Congenial Neutropenia	1	0
Griscelli	1	0
H Syndrome	1	0
Hyper IgD	1	0
IPEX	1	0
LRBA	1	0
XMEN	1	0
XLP2	1	0
Undefined	1	0
**TOTAL**	**154**	**33**

Table S2 A: Enterovirus positive cases in patients with Predominantly Antibody Deficiency (PAD) in the Phase I study.

PID types in PAD	Total no. of cases	EV positive cases
CVID	20	6
Agammaglobulinemia	16	0
XLA	16	2<br>(1 P3SL)
Hypogammaglobulinemia	9	1
TRNT1 deficiency	2	2
IgA deficiency	1	0
PIK3CD	1	0
**TOTAL**	**65**	**11**

Table S2 B: Enterovirus positive cases in patients with Combined Immuno-Deficiency during the Phase-I study.

PID types in CID	Total no. of cases	EV positive cases
SCID	14	1 (1 P1SL)
WAS	12	5 (1 P3SL)
Hyper IgM syndrome	9	4 (1 iVDPV1)
Dock 8 deficiency	6	1
AT with IgA deficiency	2	1
Ataxia Telangiectasia	2	2
CARD11 LOF	1	0
MHC Class II deficiency	1	0
ICF syndrome	2	1
**TOTAL**	**49**	**15**

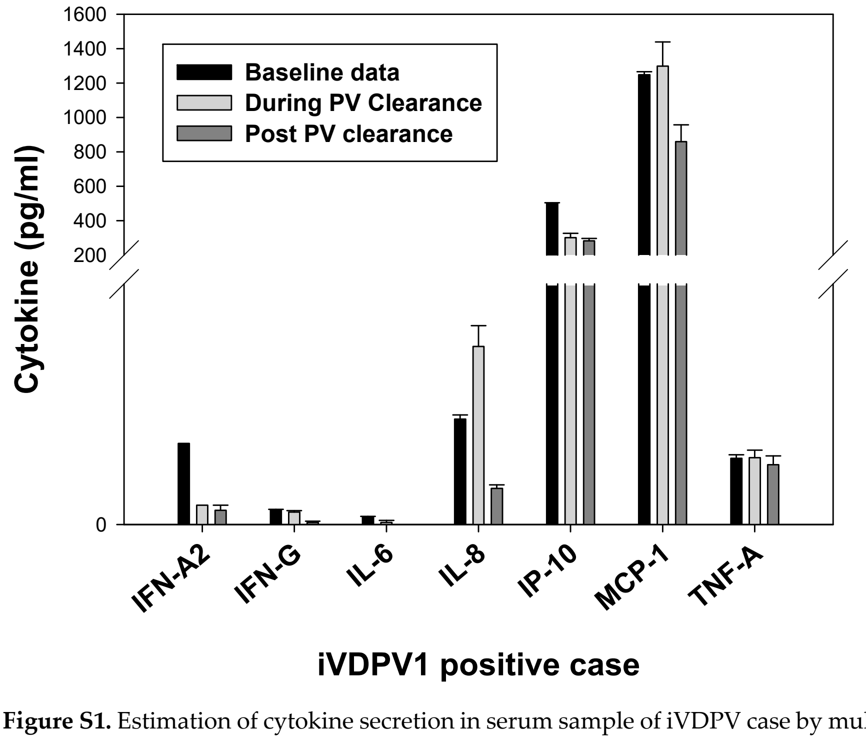

Figure S1. Estimation of cytokine secretion in serum sample of iVDPV case by multiplex ELISA: IL8 was found to be significantly high during the viral clearance as compared to the base line and post clearance period.
